# Regular rhythmic primes improve sentence repetition in children with developmental language disorder

**DOI:** 10.1038/s41539-023-00170-1

**Published:** 2023-07-10

**Authors:** Anna Fiveash, Enikő Ladányi, Julie Camici, Karen Chidiac, Catherine T. Bush, Laure-Hélène Canette, Nathalie Bedoin, Reyna L. Gordon, Barbara Tillmann

**Affiliations:** 1 Lyon Neuroscience Research Center, CNRS, UMR 5292, INSERM U1028, F-69000 Lyon, France; 2 University of Lyon 1, Lyon, France; 3 The MARCS Institute for Brain, Behaviour and Development, Western Sydney University, Sydney, Australia; 4 Department of Otolaryngology – Head & Neck Surgery, Vanderbilt University Medical Center, Nashville, TN USA; 5 Department of Linguistics, University of Potsdam, Potsdam, Germany; 6 University of Lyon 2, Lyon, F-69000 France; 7 Vanderbilt Brain Institute, Vanderbilt University, Nashville, TN USA; 8 Vanderbilt Genetics Institute, Vanderbilt University, Nashville, TN USA; 9 Vanderbilt Kennedy Center, Vanderbilt University Medical Center, Nashville, TN USA; 10 Laboratory for Research on Learning and Development, LEAD – CNRS UMR5022, Université de Bourgogne, Dijon, France

**Keywords:** Human behaviour, Language, Dyslexia

## Abstract

Recently reported links between rhythm and grammar processing have opened new perspectives for using rhythm in clinical interventions for children with developmental language disorder (DLD). Previous research using the rhythmic priming paradigm has shown improved performance on language tasks after regular rhythmic primes compared to control conditions. However, this research has been limited to effects of rhythmic priming on grammaticality judgments. The current study investigated whether regular rhythmic primes could also benefit sentence repetition, a task requiring proficiency in complex syntax—an area of difficultly for children with DLD. Regular rhythmic primes improved sentence repetition performance compared to irregular rhythmic primes in children with DLD and with typical development—an effect that did not occur with a non-linguistic control task. These findings suggest processing overlap for musical rhythm and linguistic syntax, with implications for the use of rhythmic stimulation for treatment of children with DLD in clinical research and practice.

## Introduction

Developmental language disorder (DLD) affects ~3–7% of the population and involves delayed and disordered language comprehension and/or production that cannot be attributed to peripheral deficits or global impairments in other cognitive domains^1^. Although the DLD phenotype is heterogeneous, symptoms primarily affect the domain of morphosyntax, including the use of morphological markers and complex syntax processing (encompassing the understanding and production of sentences with multiple clauses)^2–4^. Limitations in language processing result in a struggle to understand peers, teachers, and parents, and to efficiently express thoughts, which can lead to lifelong consequences in individuals’ academic and social life^5^. Effective speech-language therapy is essential to mitigate these consequences; yet DLD is greatly understudied, especially with respect to its high prevalence, compared to other neurodevelopmental disorders^6^.

Rhythmic priming is a short-term rhythmic stimulation with demonstrated benefits for grammar task performance that might be of clinical relevance to children with language impairments, and can also inform the theoretical understanding of connections between rhythm and grammar processing^7,8^. This line of research builds on previous work showing the relationship between rhythm and grammar processing in various populations^9^ and age ranges^10^. In general, priming refers to the effect of a stimulus (the prime) on the processing of a subsequent stimulus (the target). In rhythmic priming experiments, regular and irregular rhythmic (or other control) primes are presented before a set of naturally spoken sentences, and participants perform a language task on these sentences. Results show that for children with typical development (TD), with dyslexia, and with DLD, grammaticality judgments are improved after regular compared to irregular rhythms or other control conditions^7,11–15^.

The primary hypothesis underlying this paradigm is that engaging the beat-based rhythm processing system impacts subsequent language processing via shared underlying mechanisms^8^. It is supported by frameworks positing that (1) endogenous neural oscillations synchronize with the steady and consistent beat of regular rhythms and persist once the rhythm stops (in line with dynamic attending theory^16,17^), benefiting subsequent sentence processing, and (2) rhythm and language share numerous facets of neural and cognitive processing (see for example the *Processing Rhythm in Speech and Music (PRISM) framework*^8^ and^18^ for a neuroimaging meta-analysis). More broadly, rhythmic priming results fit into a research domain showing strong connections between music and language processing in the brain. Similarities between music and language in relation to syntax^19,20^, rhythm^21,22^, and auditory processing^23,24^ suggest the potential capacity for transfer effects across domains, and mounting evidence has shown that music training can causally enhance various aspects of language processing, including tracking of the speech signal^25–27^, phonological awareness^28–30^, and reading^31,32^. See refs. ^33–38^ for more general links between music and language processing and their neural correlates.

The current study focused on the rhythm-grammar link, and investigated whether rhythmic priming affects complex syntax task performance. We tested for its potential benefit on sentence repetition, a task sensitive to syntactic knowledge^39,40^ in children with DLD and TD, with clinical and theoretical aims. It is crucial to investigate the rhythm-grammar link across different language tasks: (1) not requiring a conscious reflection upon grammaticality, and (2) reflecting clinical characteristics of children with DLD. Clinically, sentence repetition tasks are sensitive for DLD diagnosis, as the repetition of grammatically complex sentences is particularly challenging for children with DLD^39^. Short-term enhancement of sentence repetition performance would suggest rhythmic priming as a valuable tool in clinical use to increase the efficacy of treatment programs, as previously suggested for syntax processing in populations with hearing loss^41^. Theoretically, assessing the rhythmic priming effect on sentence repetition would further show a benefit on grammatical processing outside of error detection, with implications for potentially shared underlying brain networks and the previously suggested rhythm-grammar processing link^10,42^.

We additionally measured individual differences in key demographic and cognitive characteristics to investigate links with the rhythmic priming effect. Chronological age was included, as grammatical sentence processing and production was expected to increase with age. Reading age was included, as previous results in TD children have shown a correlation with increased reading age and benefit of rhythmic primes on grammaticality judgements^13^. Digit span (i.e., the number of digits that could be recalled in sequence) was measured to investigate the impact of short-term memory capacity on sentence repetition, and beat-based rhythm perception abilities (measured with the beat alignment test^43^) were measured to investigate whether participants with greater rhythmic abilities might benefit more from the rhythmic primes. The goal of including these characteristics in the analysis was to establish profiles of children that could benefit the most from rhythmic priming in the present experimental paradigm, and then potentially within speech-language therapy.

## Results

### Regular rhythmic primes improve sentence repetition

French-speaking children with DLD and age-matched children with TD aged 5.4–13 years listened to regular or irregular rhythms followed by sets of six sentences. After listening to each sentence, they repeated the sentence as accurately as possible. Sentence repetitions were recorded and scored blindly offline, with possible scores of 0, 0.5, or 1, focusing on grammatical features of each reproduced utterance (see Methods for more information). A control task was run where children listened to regular or irregular rhythms before performing a visual cancellation task (cross-out as many animals as possible in a given time period).

**Fig. 1:**
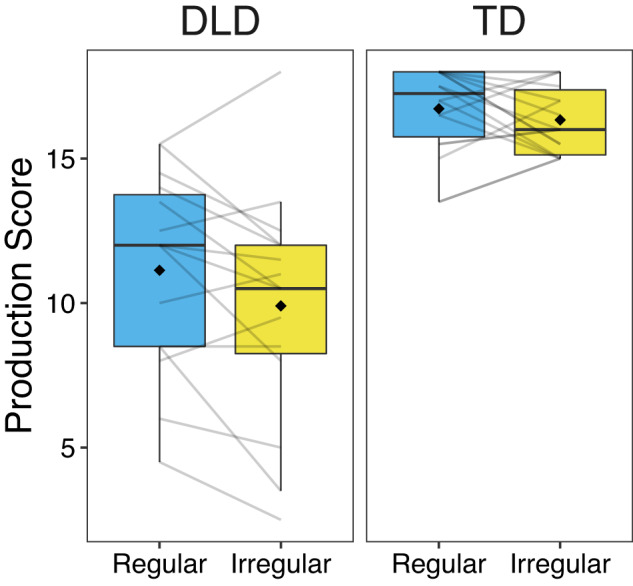
Sentence Repetition Scores. Total sentence repetition scores after regular and irregular rhythmic primes for children with developmental language disorder (DLD) and typically developing age-matched controls (TD). Each sentence repetition could receive a score of 0, 0.5, or 1. The total possible score in each condition was 18. Boxplots represent the spread of data and the median score, and the diamond represents the mean in each condition. Individual lines represent individual participant data.

To investigate whether regular rhythmic primes improved sentence repetition performance compared to irregular rhythmic primes, cumulative link mixed models were run. Prime (regular, irregular) and group (DLD, TD) were included as fixed effects, and participants and sentences as random effects (details in Methods). Overall, both prime and group had significant effects (in the expected directions) on sentence repetition scores (Fig. 1). Prime, *χ*^2^ (1) = 6.36, *p* = 0.01, AIC = 1202.2, and group, *χ*^2^ (1) = 29.81, *p* < 0.001, AIC = 1178.7, significantly improved the intercept-only model (AIC = 1206.51). The prime x group interaction, *χ*^2^ (1) = 0.39, *p* = 0.53, AIC = 1175.73 did not improve the model with prime and group as fixed effects (AIC = 1174.12). The final base model (with fixed effects of prime and group), revealed higher performance after regular, emmean = 2.67, *SE* = 0.45, 95% CI [1.78, 3.55] than irregular, emmean = 2.20, *SE* = 0.45, 95% CI [1.33, 3.08] primes, estimate = 0.46, *SE* = 0.18, *z-*ratio = 2.551, *p* = 0.01, *d* = 0.44, 95% CI around effect size [0.10, 0.79], and higher performance for children with TD, emmean = 4.16, *SE* = 0.52, 95% CI [3.13, 5.18] than children with DLD, emmean = 0.71, *SE* = 0.50, 95% CI [−0.26, 1.68], estimate = 3.44, *SE* = 0.52, *z*-ratio = 6.66, *p* < 0.001, *d* = 2.32, 95% CI around effect size [1.64, 3.00]. These results show that regular rhythmic primes significantly improved sentence repetition performance across both participant groups, extending prior work showing priming effects for grammaticality judgment tasks^7,11–13^.

**Fig. 2:**
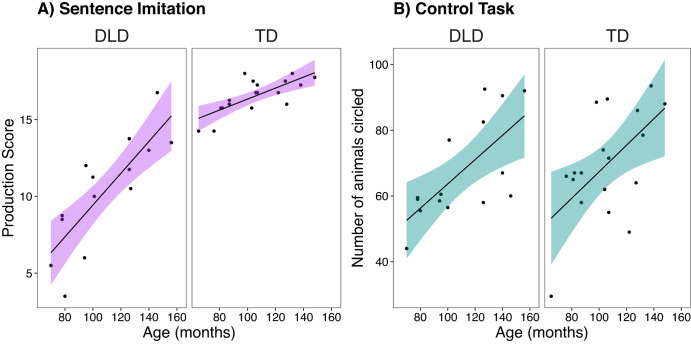
Performance Depending on Age. Performance on both tasks depending on age (months) averaged across prime condition for the developmental language disorder (DLD) and matched control (TD) groups. **A** For the sentence repetition task, sentence productions were scored for a total possible score in each condition of 18 (current data is averaged across prime conditions). **B** For the control task, the total number of animals circled was scored for a total possible score in each condition of 99 (current data is averaged across prime conditions). Regression line fitted in R with a linear model for illustration. Shaded error bars represent standard error of the mean. Individual points represent individual participants.

To investigate the potential influence of specific demographic and cognitive covariates (chronological age, reading age, digit span, rhythm perception), we added each covariate (main effect and interactions with prime, group, and prime x group) into the base model separately. The same procedure of adding and removing effects and interactions for likelihood-ratio testing was done for each covariate. (1) For chronological age, adding the fixed effect significantly improved the base model, *χ*^2^ (1) = 27.54, *p* < 0.001, AIC = 1148.6, but interactions with other factors did not, reflecting increased performance with age, trend = 1.04, *SE* = 0.16, *p* < 0.001, *z*-ratio = 6.34, *r* = 0.74, 95% CI around effect size [0.61, 0.82], see Fig. 2A. (2) For reading age, adding the fixed effect and interactions showed a significant prime x group x reading age interaction, *χ*^2^ (1) = 5.71, *p* = 0.017, AIC = 1042.1. This interaction revealed a significant trend for increased performance with reading age after regular primes in children with TD, trend = 1.26, *SE* = 0.40, *p* = 0.002, *z*-ratio = 3.13, *r* = 0.48, 95% CI around effect size [0.20, 0.66], but not after irregular primes in children with TD (*p* = 0.28) and not in children with DLD after regular (*p* = 0.35) or irregular (*p* = 0.10) primes. Note that a reduced base model was made for this analysis, since three children (two with DLD and one with TD) had missing reading age data as they were not yet able to read. Finally, the model did not significantly improve when adding (3) age-adjusted digit span scores, *χ*^2^ (4) = 5.01, *p* = 0.29, AIC = 1177.1, or (4) age-adjusted rhythm perception scores, *χ*^2^ (4) = 1.51, *p* = 0.83, AIC = 1180.6.

These results show that general performance on the task increased with age (see Fig. 2A), but was not influenced by digit span or beat-based rhythm perception scores. Importantly, the rhythmic priming effect (i.e., the benefit of the regular compared to irregular rhythmic primes) was not influenced by age or individual differences in cognitive abilities in children with DLD, suggesting benefits across ages and cognitive abilities. However, higher reading age was associated with an increased priming effect in children with TD, supporting previous results^13^. The effect sizes of group and age were very large, showing that these predictors strongly influenced sentence repetition, as might be expected. The effect size of prime in comparison was small (though approaching a medium effect size); however, it emerged across the full sample regardless of age, suggesting potential for clinical applications.

### Regular rhythmic primes do not improve control task performance

For the control task, the same analyses were run to ensure that the rhythmic priming effect was not a more general arousal or motivational effect. A generalized linear mixed model (poisson distribution) was run on count data (number of correctly crossed-out animals). Compared to the intercept-only model (AIC = 1155.1), none of the additions: prime, *χ*^2^ (1) = 0.01, *p* = 0.91, AIC = 1157.1, group, *χ*^2^ (1) = 0.09, *p* = 0.76, AIC = 1157.0, or prime x group interaction improved the model, *χ*^2^ (1) = 0.08, *p* = 0.77, AIC = 1159.0. Only the main effect of chronological age improved the model, *χ*^2^ (1) = 15.56, *p* < 0.001, AIC = 1145.5, reflecting increased performance with age, trend = 0.15, *SE* = 0.03, *p* < 0.001, *z*-ratio = 4.45, *r* = 0.61, 95% CI around effect size [0.40, 0.74], see Fig. 2B. No other covariates revealed significant main effects or interactions. These results showed no difference in performance on the visual cancellation task depending on prime or group, thus ruling out that rhythmic priming appears due to general arousal or motivational effects (supporting previous work^12,14^).

## Discussion

Our findings showed improved sentence repetition performance after regular compared to irregular rhythmic primes in children with DLD and age-matched TD controls. The control task confirmed that the priming effect was not a general arousal, motivational, or distraction effect of the rhythmic primes. The presence of rhythmic priming in grammaticality judgment tasks in previous studies^7,11–15,44,45^ and in the current sentence repetition task, paired with the lack of this effect in tasks without a grammar component here and in previous work^12,14^ suggests a specific benefit of regular rhythms on grammar processing.

The current findings converge with (1) neuroimaging results showing some degree of overlap between networks recruited for rhythm and grammar processing^18^ and (2) behavioral evidence showing associations between individual differences in rhythm processing and processing of sentences with complex syntactic structures (e.g. refs. ^10,46^). Processing hierarchical structures (including prediction) have been proposed to play a role in acquisition and usage of rhythm and grammar skills^18,47^. Our results are thus consistent with the hypothesis that shared mechanisms may underly rhythm and grammar processing in the brain^8,18,42^. The present findings also fit into previous research showing associations between multiple aspects of music and language abilities (see refs. ^33,48–50^ for review), as well as the more general view that non-linguistic processes play a role in language development (e.g. ref. ^51^).

Based on previous evidence on relations between sentence processing and executive functions (e.g., ref. ^52^) and between musical processing and executive functions^53^, one could argue that the rhythmic priming effect might be mediated by executive functions in the current study (i.e., regular primes improved executive functions, which improved sentence repetition performance). However, this explanation is unlikely as rhythmic priming did not facilitate performance on non-grammatical tasks^12,14,54^, including an executive function task^12^, in the current study or in previous studies. An avenue for further research could be to clarify the interplay between executive functions, rhythm, and grammar abilities in general, and in the rhythmic priming context in particular.

Future research will allow for furthering our understanding of typical as well as pathological brain functioning in musical rhythm and linguistic grammar processing, which will open also to rehabilitation and training perspectives. Based on the current results and previous work showing a link with reading age and the efficacy of regular rhythmic primes in TD children^13^, it will be particularly promising to investigate links between reading age and efficacy of rhythmic priming, as well as the mechanisms underlying this connection. The finding that beat-based rhythm perception performance (BAT) did not influence the rhythmic priming effect might suggest that rhythmic priming works across different levels of rhythmic skills (at least in relation to beat-based rhythm perception, see ref. ^55^). This finding could indicate that participants do not need a certain level of rhythmic ability to be able to benefit from rhythmic priming, which would be promising for training and rehabilitation. Although here we did not find a significant difference in rhythm perception (as measured by the BAT, *p* = 0.057) between the TD and the DLD group, the TD group performed better on the BAT than the DLD group did overall (see previous reports of poor performance on rhythm tasks in DLD^11,56,57^). Future research can further investigate potential differences across different aspects of rhythmic competencies^55^, and individual differences in rhythmic reward (Fiveash et al.^58^) in children with DLD compared to controls, and their link with rhythmic priming potential. More globally, future research testing the effect of rhythmic priming on other language tasks and with neural methods could help to better understand the exact mechanisms underlying the rhythmic priming phenomenon, with its strengths and limitations.

The current results have clinical implications for the use of rhythm in speech-language therapy to boost and train the processing of complex grammatical structures. Sentence repetition is a valuable indicator of DLD, as performance appears to tap into syntactic skills as well as lexical knowledge and memory^39,40^. Combined with previous beneficial effects of rhythmic priming for grammaticality judgment tasks, and the current finding that digit span did not influence performance, overall findings suggest a benefit of rhythmic stimulation on syntactic processing. Therefore, using rhythmic priming during clinical sessions may help to enhance the learning of syntax and performance on syntactic tasks, which may then transfer outside of the session (see^41^ for an example of how rhythmic priming can be used in long-term training sessions of speech therapy). The efficacy of using rhythmic priming with grammar tasks targeting other areas of difficulty in DLD, such as the use of morphological markers, merits further research, as do individual differences in who can benefit the most from rhythmic priming, both in research settings and in speech-language therapy.

There are two potential limitations that should be acknowledged within the current experiment. First, our sample size of 15 children with DLD and 18 typically developing children could be considered as being relatively small, with an age range spreading from 5.4 to 13 years. However, as recruiting clinical populations is difficult, the current sample size is commonly observed in related studies investigating children with DLD^3,11,59–62^, given the estimated prevalence and potential underdiagnosis in the population^6^. Although less common, previous research has also tested DLD groups with a wide age range^3,7,61^. Second, the performance of the TD group was overall rather high, leaving limited room for improvement following regular rhythmic primes compared to irregular rhythmic primes. While our results still show evidence for a priming effect across both the TD and the DLD groups, a task that is more challenging for the TD group might potentially allow for better expression of the priming effect in this group. Our present findings provide evidence that regular rhythmic primes improve sentence repetition compared to irregular rhythmic primes across groups and ages. Future research should replicate this finding in a larger sample, with potentially more difficult items for the TD children, and further investigate the potential developmental progression of the priming effect.

Although the current study was designed as a within-subjects experimental paradigm and not a randomized clinical trial, the results are in line with previous music intervention studies that used rhythm as a component of treatment successfully targeting language outcomes (e.g., ref. ^32^). Importantly, as sentence repetition is a central area of difficulty for children with DLD, the current results suggest that using regular rhythms to boost syntactic processing during speech-language therapy sessions could be a promising avenue to investigate further (see ref. ^41^) in future clinical research. More generally, the myriad of associations between multiple aspects of music and language processing (e.g., refs. ^5,8,33,48,63^) make music (and music rhythm in particular), a promising tool to be used in speech therapy, as well as for the scaffolding of language abilities in typically developing populations.

## Methods

### Participants

Participants were 15 children with DLD (7 girls, 8 boys) and 18 age-matched control children (11 girls, 7 boys), between 5.4 and 13 years, all native speakers of French. All children in the DLD group had an official diagnosis of DLD from a certified speech-language therapist, and none of the included children with TD had any reported language problems. Children with intact hearing, without any genetic or neurological disorders reported by the parent, and a non-verbal IQ above 78 measured by the Test of Nonverbal Intelligence (TONI-4^64^), were included in the study. Groups were matched for chronological age (see Table 1, no significant difference in age between the DLD and TD groups), and the mean (absolute) difference between each participant of an age-matched pair was 3.83 months, *SD* = 4.72). Data from an additional nine children were collected, but excluded due to a technical error (four children with DLD, one with TD) or failing to meet inclusion criteria (four children with TD).

**Table 1:** Group performance (mean, SD) on the screening and experimental measures for children with developmental language disorder (DLD) and typical development (TD).

	Measure	DLD Mean (SD)	TD Mean (SD)	*p*-value
Screening Measures	Age (months)	110.47 (28.31)	105.44 (23.29)	0.58
	CELF repetition (standard)	6.73 (2.15)	12.83 (1.82)	<0.001*
	CELF elaboration (standard)	7.00 (2.20)	12.17 (1.82)	<0.001*
	Phonological perception (z-score)	−0.56 (1.32)	0.78 (0.98)	0.002*
	Non-word repetition (z-score)	−4.29 (2.90)	0.16 (0.84)	<0.001*
	Non-verbal intelligence (index)	100.67 (9.01)	114.5 (7.76)	<0.001*
Experimental Measures	Reading age (z-score)	−1.21 (1.23)	0.38 (1.22)	0.001*
	Digit Span (age-corrected)	−1.83 (1.67)	1.52 (2.05)	<0.001*
	BAT (age-corrected)	−0.38(1.15)	0.32 (0.88)	0.057

See methods for details on all analyses. All tests reflect age-normed scores based on published norms, except for digit span and the beat alignment test (BAT) which were age-corrected. (see Testing and Covariates section below), For testing between groups, independent samples *t*-tests were run, with equal variance assumed true if Levene’s test was passed (age, CELF repetition, CELF elaboration, phonological perception, non-verbal intelligence, reading age, digit span, BAT) and equal variance assumed false if Levene’s test was not passed (non-word repetition). All tests were two-sided. Asterisks refer to significant differences between groups.

All children with DLD (except one, see below) performed below standardized cut-offs on at least one of the following tasks: (1) Sentence Repetition and (2) Sentence Elaboration subtests from the French version of the Clinical Evaluation of Language Fundamentals (CELF-5^65^; age-normed cut-off = 7 in each task) or (3) the non-word repetition test from the Batterie Analytique du Langage Écrit^66^ (>2 SD below the norm). These tasks are typically used as clinical markers for DLD^67,68^. One older child with DLD performed just above the cut-off on the two CELF tests (with a standardized score of 8; 25th percentile), and below the norm (though within 2 SD) on the non-word repetition test, so was still included. All included TD children performed above these cut-offs. On average, the DLD group performed significantly lower than the TD group for all tasks (see Table 1). Children with DLD also performed significantly lower than children with TD on a phonological perception task^69^ (see Table 1). The study was approved by the national ethics committee (Comité de Protection des Personnes; CPP) and was run in accordance with the Declaration of Helsinki and CPP regulations. Parents provided written informed consent and children provided verbal assent after being provided with an illustrated information sheet.

### General design

Children came to the lab two times to complete all tasks. The sentence repetition task and the cancellation task were performed in different visits, and order was counterbalanced across children. The additional tasks testing for language and cognitive skills were presented in a fixed order and interspersed with experimental tasks. The beat alignment test (BAT) for rhythm perception was always completed at the end of the second visit so that it did not interfere with the priming tasks. Children also completed further behavioral tasks outside the scope of the current study. The sentence repetition priming task and the BAT, as well as the prime presentation for the visual cancellation task were programmed and presented in E-Prime 2.0. Children received a small gift at the end of each experimental session.

### Sentence repetition priming task

#### Rhythmic stimuli

Rhythmic primes were temporally either regular or irregular and were selected from a larger set used in Fiveash et al.^13^. Three different regular rhythms and their irregular versions were used. The regular rhythms were composed by a musicologist and consisted of a regular beat (500 ms inter-beat-interval, 120 beats per min) in a 4/4 meter. The rhythms consisted of various percussion instruments and electronic sounds from virtual studio technology instrument timbres (e.g., cymbal, tom-tom, snare drum, bass drum) to increase musical and acoustic complexity. Irregular rhythms consisted of the same notes and durations as the regular rhythms but randomized so that it was not possible to perceptually extract an underlying beat or a regular metric. Rhythmic stimuli can be accessed at https://osf.io/msbn4/.

#### Sentence stimuli

Thirty-six sentences were created: twelve sentences with subject-relative clauses (e.g., Elle est la femme qui a vu Frank dehors [This is the woman who saw Frank outside]), 12 sentences with object-relative clauses (C’est le chat dont Tom s’est caché hier [That’s the cat that Tom hid from yesterday]), and 12 simple filler sentences without a relative clause (e.g., Les enfants jouaient dans le parc [The children played in the park]). Subject-relative clauses had an average of 13.08 (*SD* = 2.35) syllables, 11.50 (*SD* = 1.38) words; object-relative clauses had an average of 12.58 (*SD* = 1.78) syllables, 10.92 (*SD* = 1.16) words; and filler sentences had an average of 6.0 (*SD* = 1.60) syllables, 4.92 (*SD* = 1.24) words. Sentences were recorded by a native French-speaking woman who produced the sentences at a comfortable rate with a neutral tone. The intensity of the auditory stimuli was normalized across all primes and sentences. Sentence stimuli can be accessed at https://osf.io/msbn4/.

#### Design

The sentence repetition priming task consisted of six blocks. For each block, a 32-s regular or irregular rhythmic prime was presented to the child over headphones while the experimenter was listening to masking stimuli (over headphones). Immediately following the rhythm, six sentences were played, and the child repeated each sentence directly after it finished playing. Sentences presented within a block were the same across participants (with two subject-relatives, two object-relatives and two simple sentences without a relative clause), but in a different randomized order for each participant. Regular and irregular prime blocks were alternated, with different prime rhythms pseudo-randomized across participants so that the regular and irregular versions of the same rhythm could not appear consecutively. The starting rhythm (regular or irregular) and the sentences paired with the regular and irregular primes were counterbalanced across participants. This presentation ensured that each sentence appeared an equal number of times in a regular and irregular condition across participants. The sound level of presentation was set to a comfortable loudness level.

#### Procedure

Children were tested individually in sound-proof booths. The experimenter explained that the children were going to play an imitation game, and that they would first hear some music from the computer, then some sentences, and that they would have to repeat them out loud in a clear voice. Children heard three practice sentences, and were given feedback on their repetitions (e.g., Great! You said exactly what you heard! Or Almost! The person said*…*). Then they were told the real game would begin, and that they would hear music followed by six sentences that they had to repeat out loud. They were reminded to listen carefully to the music and sentences. All stimuli were presented through headphones. Children gave verbal responses throughout the task, and the experimenter advanced the experiment after each trial once the child had repeated the sentence. Responses were recorded with an external audio recorder (ZOOM H1n). To motivate the children, they selected a laminated character that they moved along after each block to visualize their progress. At the end of the experiment, they received a sticker to add to their sticker sheet. The task took ~13 min.

#### Scoring

A trained researcher with expertise in speech production and perception research transcribed, coded, and scored the child-produced sentences based on audio recordings (with the written target sentence for comparison). All word or morpheme omissions, additions, transpositions, and substitutions were coded, and each sentence was scored based on the type of errors in the sentence. Scoring was adapted from Diessel and Tomasello^70^ with the aim to create a system specifically targeting grammar skills. For each sentence production, children received 0 points if they omitted a syntactically obligatory word from the sentence or if the main structure of the sentence changed (e.g., producing a sentence with a subject-relative clause instead of an object-relative clause); 0.5 points if the error was grammatical but did not obscure the semantic content nor syntactic structure (e.g., determinant omission, substitution of preposition, morphological error such as a tense marker at the end of a verb; and 1 point if they did not make an error or if the error was not grammatical in nature (e.g., lexical error with substitution of one word by another, saving syntactic consistency, or omission of an unnecessary word). For example, we did not penalize word substitutions so long as the grammatical structure of the sentence was correct. Thirteen out of the 33 recordings (experimental sessions of six DLD and seven TD participants) were randomly selected to be transcribed, coded, and scored by a second coder (a speech therapist who was also trained in the coding scheme described above) to test the reliability of the first coder. We calculated percentage agreement out of the 36 sentences and found an average inter-rater-reliability of 0.91 for the DLD children and 0.99 for the TD children, so we maintained the scores of the first scorer. During transcription, coding and scoring of the sentences, scorers were blind to the prime condition the sentences belonged to.

### Visual cancellation priming task

The visual cancellation priming task also consisted of six blocks, with a rhythmic prime (regular or irregular) followed by one worksheet of the visual cancellation task. Worksheets were adapted from the cancellation subtest of the Wechsler Intelligence Scale for Children^71^ that contained two different sheets, each including various objects and animals, with a total of 32 animals on each sheet. To be able to present six different sheets in the priming task (one for each block), four additional sheets were created by adapting the original sheets. One of the sheets created this way included 35 animals. The order of sheets was the same for each child across the experiment. Each of the six sheets appeared an equal number of times in regular and irregular blocks across children as starting rhythm was counterbalanced. After each prime, children crossed-out as many animals as they could in 23 s. After the experimental session, the experimenter counted the total number of animals crossed out and subtracted any additional objects that were crossed out to obtain a final performance score after each prime condition and which was used for the analyses.

### Reading task

Reading age was measured by reading aloud a short text called “Monsieur Petit” from the “Évaluation de la lecture en fluence” test (E.L.FE, *Evaluation of Reading Fluency*)^72^. Children had 1 min to read as much as they could from a one-page text telling a story. The number of errors made were subtracted from the total number of words read to provide a total score, which was then indexed against age-norms published in the E.L.FE to provide a z-score depending on the mean and standard deviations of each age group.

### The beat alignment test

The BAT is a measure of beat-based rhythm perception that was originally proposed by Iversen and Patel^43^. The current implementation included a child friendly cover-story and the same stimuli as used in Fiveash et al.^55^, which included 24 musical excerpts from the BAASTA^73^, presented at a tempo with an inter-beat-interval of 600 ms. Each musical excerpt contained a tone played with a triangle timbre that started ~3–4 s after the beginning of the excerpt. The triangle tone was either on the beat (aligned) or off the beat (either phase or period misaligned). In phase misaligned trials, the triangle tone was shifted 33% before or after the beat in the same tempo. For period misaligned trials, the triangle tone was presented consistently 10% slower or faster than the beat. Children were told that they were in the jury to decide which puppet would be in a rock band, and that they had to decide if each puppet was playing a cymbal on the beat or off the beat. The beat was described as the feeling of something regular in music, like when you clap your hands or move to the beat of the music. Children heard examples of on-the-beat and off-the-beat trials, and then they had practice trials where they received feedback. Chronological age-adjusted *d’* scores were calculated and used in the analyses (see below).

### Digit span

The number recall task from the Kaufman Assessment Battery for Children (KABC-II^74^) was used to measure digit span (maximum score was 20). Chronological age-adjusted scores were calculated and used in the analyeis (see below).

### Analyses

#### Sentence repetition priming task

The sentence repetition task was analyzed using cumulative link mixed models (CLMM) to model fixed and random effects on ordinal data (*clmm* function from the ordinal^75^ package in R^76^). Cumulative link mixed models were used because the data were categorical, with an ordered but not necessarily linear relationship between each score (0, 0.5, 1). These models allow for the inclusion of both fixed and random effects, to increase power and control for participant and item effects. The random effects structure included random intercepts for participant and item (suggested in Baayen et al.^77^). Effects of group and prime were contrast-coded (−0.5, 0.5) so that comparisons were made to their mean value rather than holding one condition as a baseline. A logit link was used in all models, making the CLMM model equivalent to a proportional odds mixed model.

#### Visual cancellation priming task

For the visual cancellation control task, a generalized linear mixed model using a poisson distribution (for the count data) was run using the *lme4* package^78^ in R Studio (comparable to the cumulative link model but for count data). Only the participant random effect was included in the model as there was only one sheet after each given prime excerpt (with a total of three sheets for each prime condition).

#### Testing and covariates

Likelihood ratio tests were used to compare models with and without different fixed effects using drop1 and add1 methods. Directions of fixed effects were investigated using *emmeans*^79^. Significant effects that included covariates were investigated using *emtrends* to model the covariate data direction. Effect sizes were calculated with the package *effectsize*^80^ based on the z-ratio and sample size reported by *emmeans*. Cohen’s *d* and Pearson’s correlation coefficient (*r*) are reported. Cohen’s *d* effect sizes were interpreted according to the guidelines in^81^ and the *effectsize* package: *d* < 0.2 = *very small*; 0.2 <= *d* < 0.5 = *small*; 0.5 <= *d* < 0.8 = *medium*; and *d* >= 0.8 = *large*. For *r*, 0.1 <= *r* < 0.2 = *small*; 0.2 <= *r* < 0.3 = *medium*; 0.3 <= *r* < 0.4 = large; and *r* >= 0.4 = very large^80^. We additionally calculated the 95% confidence interval around the effect sizes to estimate confidence in our effect sizes^82^.

To analyze the effects of chronological age, reading age, digit span, and rhythm perception, each covariate was first scaled and normalized. As the rhythm perception task (BAT) and the digit span task were not age-normed, we extracted the residuals from a regression analysis that was run to predict BAT/digit span from chronological age and used these age-adjusted scores to measure the pure effect of these covariates. Covariates were added separately (i.e., only one covariate in each model) into the base models defined above (with prime and group as fixed effects). For each covariate, their main effect, interaction with prime, interaction with group, and interaction with prime and group were added. Using the same add1 and drop1 methods as described above, the likelihood ratio tests informed whether each covariate and their interactions significantly improved the base model.

#### Additional analyses

It could be argued that language processing might rapidly develop between the ages of 5–7 when children enter school and learn more complex grammatical structures and reading. We thus ran supplementary analyses on the sentence repetition data, restricted to children 7 years and older (11 DLD and 14 TD) and observed the same pattern of results as for the full groups.

## Data Availability

All stimuli, data, and scripts used in the analyses can be found at https://osf.io/msbn4/.

## Code availability

The R scripts we used for data analyses are available at https://osf.io/msbn4/.
